# Public Perceptions of the Australian Health System During COVID‐19: Findings From a 2021 Survey Compared to Four Previous Surveys

**DOI:** 10.1111/hex.14140

**Published:** 2024-07-11

**Authors:** Louise A. Ellis, Genevieve Dammery, James Gillespie, James Ansell, Leanne Wells, Carolynn L. Smith, Shalini Wijekulasuriya, Jeffrey Braithwaite, Yvonne Zurynski

**Affiliations:** ^1^ Centre for Healthcare Resilience and Implementation Science, Australian Institute of Health Innovation Macquarie University Sydney New South Wales Australia; ^2^ NHMRC Partnership Centre for Health System Sustainability Sydney New South Wales Australia; ^3^ Sydney School of Public Health, Menzies Centre for Health Policy The University of Sydney Sydney New South Wales Australia; ^4^ Consumers Health Forum of Australia Canberra Australian Capital Territory Australia

**Keywords:** Australia, consumer sentiment, COVID‐19, healthcare system, public perceptions

## Abstract

**Background:**

This study examines the perceptions of the Australian public canvassed in 2021 during the COVID‐19 pandemic about their health system compared to four previous surveys (2008, 2010, 2012 and 2018).

**Methods:**

In 2021, a nationwide online survey was conducted with a representative sample of Australians (*N* = 5100) recruited via market research panels. The results were compared to previous nationwide Australian survey samples from 2018 (*N* = 1024), 2012 (*N* = 1200), 2010 (*N* = 1201) and 2008 (*N* = 1146). The survey included questions consistent with previous polls regarding self‐reported health status and overall opinions of, and confidence in, the Australian health system.

**Results:**

There was an increase in the proportion of respondents reporting positive perceptions at each survey between 2008 and 2021, with a significantly higher proportion of respondents expressing a more positive view of the Australian healthcare system in 2021 compared to previous years (*χ* ^2^(8, *N* = 9645) = 487.63, *p* < 0.001). In 2021, over two‐thirds of respondents (*n* = 3949/5100, 77.4%) reported that following the COVID‐19 pandemic, their confidence in the Australian healthcare system had either remained the same (*n* = 2433/5100, 47.7%) or increased (*n* = 1516/5100, 29.7%). Overall, respondents living in regional or remote regions, younger Australians (< 45 years) and women held less positive views in relation to the system. In 2021, the most frequently identified area for urgent improvement was the need for more healthcare workers (*n* = 1350/3576, 37.8%), an area of concern particularly for Australians residing in regional or remote areas (*n* = 590/1385, 42.6%).

**Conclusions:**

Irrespective of disruptions to the Australian healthcare system caused by the COVID‐19 pandemic, Australians' perceptions of their healthcare system were positive in 2021. However, concerns were raised about inadequate workforce capacity and the cost of healthcare, with differences identified by age groups and geographical location.

**Patient or Public Contribution:**

Health consumer representatives from the Consumers Health Forum of Australia contributed to the co‐design, deployment, analysis and interpretation of the results of this survey. J.A. and L.W. from the Consumers Health Forum of Australia contributed to the development of the paper.

## 1 Introduction

The emergence of the COVID‐19 pandemic was profoundly influential on the general public's perception of disease and healthcare globally, with the pandemic affecting both the delivery of healthcare and people's experiences of receiving healthcare [1]. Research has suggested that the effects of the pandemic have changed public opinion and behaviours in relation to the healthcare system, including impacts on access to care and rapidly changing policies [1].

Government responses in relation to the pandemic varied substantially across countries and sectors. Australia is amongst a small number of countries that pursued a national ‘aggressive suppression’ strategy involving the early application of strict policy decisions relating to ‘containment and closure’ [2] to avoid the consequences of the large waves of infections seen in many other parts of the world. Australia also adopted a broad testing approach, which allowed for the identification of asymptomatic cases, good contact tracing and mandatory isolation for those testing positive [2]. Research conducted in 2020 identified that the adoption of early stringency measures in tandem with broad testing approaches effectively blocked the spread of the virus in the early stages of the pandemic, with Australia recording one of the lowest rates of COVID‐19 cases and deaths [3]. Further, during the pandemic, the Australian public showed high levels of trust in the federal government and were, by and large, compliant with the key policy measures initiated by the government [4]. Although the impacts of government policies and public perceptions on healthcare delivery, utilisation and health outcomes are apparent; the longer‐term impact on health outcomes is unclear and has been proposed as an important topic of ongoing study [5].

During the first year of the pandemic, the focus of many countries was on facilitating acute medical care, especially care directly related to COVID‐19 infection, with most scheduled and preventative care being cancelled or postponed [6]. However, there were also reports of increased healthcare avoidance by the general public, irrespective of the country's COVID‐19 incidence rate, prompting further research into the underlying causes [7, 8]. Notably, along with demographic and health‐related factors, low public confidence in health systems' responses to the pandemic has been identified as a significant predictor for healthcare avoidance [7], highlighting the importance of monitoring public perceptions of the healthcare system, especially during pandemics and global health crises.

Surveys have long been used as a ‘barometer’ to identify the general public's perceptions of their healthcare system and can be used to drive changes in policy and systems. Since 2018, the Ipsos Global Health Service Monitor survey has been conducted annually in 30 countries, including Australia, to identify public health concerns and views about their country's health system [9]. In the 2022 Ipsos survey, 61% of respondents globally agreed that their system is ‘overstretched’, and for the first time ‘not enough staff’ was ranked as the joint top main challenge facing their country alongside treatment ‘waiting times’, followed by cost. The Commonwealth Fund has also regularly surveyed country‐level perspectives of health systems in 12 high‐income countries (now 20 countries), including Australia [10]. Findings from surveys conducted by the Commonwealth Fund and comparisons with international data from the World Health Organization (WHO), the Organisation for Economic Cooperation and Development (OECD) and the European Observatory on Health Systems and Policies have been used to inform health system policy reforms in the United States both during COVID‐19 and prior [11].

In a similar way, surveys conducted with the Australian public have been used as an indicator to reflect the state of the functioning of the health system, with significant implications for healthcare policy and practice [12]. In the 2021 survey of the Commonwealth Fund, the overall performance of the Australian healthcare system was ranked third of 11 high‐income countries and ranked eighth for providing affordable, timely access to care [10]. In our previous national survey conducted in 2019, Australians expressed concerns about access and affordability of healthcare, especially among people with chronic conditions [13]. This is despite the Australian federal government funding health coverage for all Australians through the universal public health insurance scheme, Medicare [14], which provides free or subsidised access to medical and public hospital services.

Medicare is funded by the Australian Government through taxation revenue, including a Medicare Levy on taxable income. A parallel Pharmaceutical Benefits Scheme, also funded by the Australian government, provides Australians with subsidised access to medicines [15]. Australians can also purchase supplementary private health insurance to pay for private hospital care, dental services and other ‘extras’ [16]. Australians purchasing private health insurance has continued to grow since the onset of COVID‐19, with 55% of Australians currently having private health insurance [17]. In 2023, 15% of all expenditure on health care was paid directly by healthcare consumers in the form of out‐of‐pocket expenses. Although this is lower than many other high‐income countries, there is growing concern around increasing out‐of‐pocket expenses for vulnerable groups, such as socioeconomically disadvantaged and older Australians [18].

Similar to other countries, the Australian healthcare system has been significantly disrupted by the COVID‐19 pandemic, leading to the Australian government establishing the Strengthening Medicare Taskforce to improve equity of access to primary care, encourage multidisciplinary team–based care and provide a roadmap for sustainable reform processes [19]. Although there have been public calls for more healthcare workers before 2020 [12], the pandemic required a rapid influx into the health workforce, with the Australian Government introducing the COVID‐19 Surge Workforce Program in 2022 to boost healthcare workforce numbers [20].

Increasing the understanding of the Australian public's preferences and expectations of their healthcare system will not only enhance existing national surveys (e.g., Australian Bureau of Statistics [ABS] National Health Survey [21]) and international surveys (e.g., the Ipsos Global Health Service Monitor survey [9]) but also inform health policies and plans based on the view of the public who are end‐users of the healthcare system. Indeed, the need for robust longitudinal studies about perceptions of healthcare among the adult Australian population has also been highlighted as a research priority [12].

The objective of this study was to identify the views held by the Australian public in 2021, at a time when the COVID‐19 pandemic was still very present, and examine broad differences since 2008, spanning a 13‐year period. Public sentiment was examined cross‐sectionally over five time points, by comparing the 2021 Australian Health Consumer Sentiment Survey [22] with the 2018 Australian Health Consumer Sentiment Survey [12], and three national health surveys undertaken by the Menzies Centre for Health Policy and the Nous Group in 2008 [23], 2010 [24] and 2012 [25].

## 2 Methods

The methodology for this study is similar to that used in other analyses of the 2018 and 2021 Australian Health Consumer Sentiment Survey [12, 13, 22].

### 2.1 Participants

Australian adults (aged ≥ 18 years) were recruited via a leading international digital data collection company, Dynata, with over 200,000 panellists registered in Australia. In 2021, Dynata was engaged to recruit 5000 online survey participants, adhering to representative quotas for age, gender and geographical location. Potential participants were contacted by Dynata from September to October 2021 via email and invited to take part in the online survey. All participants provided informed consent before taking part in the survey. Ethics approval was granted by the Macquarie University Human Research Ethics Committee (Ref. No.: 5201836705403).

### 2.2 Survey

The online survey was co‐designed with consumer representatives from the Consumers Health Forum of Australia. Additional feedback to the survey was also provided by the Australian Government Department of Health. As reported elsewhere, the survey included a total of 67 questions [12, 13]. The focus of this paper is on survey items that were consistent with those asked in the 2018 Australian Health Consumer Sentiment Survey [12, 13], and the three biannual Menzies‐Nous Australian Health Surveys [23, 24, 25], providing a basis for comparison. Consistent items included self‐reported health status, overall views of the Australian health system and confidence in the Australian health system. Refer to our previous paper for detailed information regarding these items [12]. Two additional items specific to the 2021 survey are also reported here as they relate to public perceptions of the Australian health system in the context of COVID‐19, described below. The results of the remaining items not reported in this paper have been published elsewhere [26, 27].

### 2.3 Additional Items Specific to the 2021 Survey

The first of two questions asked participants whether their confidence in the Australian healthcare system has changed since the COVID‐19 pandemic. For this question, participants provided their responses on a three‐point Likert scale (1 = *decreased*; 2 = *stayed the same*; 3 = *increased*). The second question asked participants to select the area of the healthcare system that ‘needs improvement most urgently’. The first set of response options was based on our previous 2018 survey (e.g., better access to care; better quality of care; more doctors, nurses and other health workers), with the addition of two COVID‐19–specific options (i.e., communication about COVID‐19 symptoms and testing; and communication about COVID‐19 vaccines). Direct comparisons of these additional COVID‐19 response options with data from previous years are not appropriate.

### 2.4 Data Analysis

As in our previous analyses [12, 13, 22], survey data were post‐weighted to reflect the respective ABS estimates [12, 13, 22, 28, 29, 30] of the population according to age, gender and state using a survey raking technique via the anesrake package in R [31]. Consistent with our previous studies [12, 22], postcode data were mapped to the Australian Statistical Geography Standard (ASGS) and aggregated into a dichotomous variable being created: major city and regional/remote. Age was aggregated into four groups: 18–24, 25–44, 45–54 and > 65 years. Consistent with our previous analyses [12], linear regression was used where categorical variables had five or more levels [32] and chi‐square (*χ* ^2^) was used to examine categorical variables for which there were less than five levels. Dummy variables were developed to examine the categorical variables of age and survey year to assist with interpretation (see Appendix S1). All data transformations and analyses were conducted using IBM SPSS Statistics Version 29.0 [33] and a *p* value of 0.001 was used for statistical significance.

## 3 Results

### 3.1 Characteristics

A total of 5100 Australians participated in the 2021 Australian Health Consumer Sentiment survey. Participants ranged in age from 18 to 99 years (*M* = 46.8, SD = 17.6). A summary of participant demographics from 2021 is presented in Table 1, together with a comparison from the previous 2018 Australian Health Consumer Sentiment survey and the three previous 2012, 2010 and 2008 Menzies‐Nous Australian Health Surveys [23, 24, 25].

**Table 1:** Study participant characteristics across the five surveys.

Characteristics	2021, *n* a (%)^b^	2018, *n* a (%)^b^	2012, *n* a (%)^b^	2010, *n* a (%)^b^	2008, *n* a (%)^b^
Overall		1024	1200	1201	1146
Gender					
Men	2475 (49.0)	432 (49.0)	539 (49.0)	540 (49.0)	420 (49.0)
Women	2576 (51.0)	592 (51.0)	661 (51.0)	661 (51.0)	726 (51.0)
Age					
18–24 years	614 (12.0)	68 (12.0)	116 (12.0)	104 (12.0)	72 (12.1)
25–44 years	1853 (36.3)	352 (37.0)	379 (37.0)	397 (38.0)	332 (38.4)
45–64 years	1589 (31.2)	383 (32.0)	479 (33.0)	504 (33.0)	492 (34.3)
65+ years	1043 (20.5)	221 (19.0)	226 (18.0)	196 (17.0)	242 (15.2)
State					
ACT	86 (1.7)	9 (2.0)	20 (2.0)	20 (1.7)	34 (2.0)
NSW	1623 (31.8)	330 (32.0)	396 (32.0)	397 (33.2)	360 (33.0)
NT	49 (1.0)	2 (1.0)	11 (1.0)	11 (1.0)	17 (1.0)
QLD	1033 (20.3)	218 (20.0)	233 (20.0)	233 (19.2)	233 (20.0)
SA	351 (6.9)	83 (7.0)	92 (7.0)	92 (7.5)	99 (7.0)
TAS	108 (2.1)	22 (2.0)	29 (2.0)	29 (2.2)	5 (2.0)
VIC	1319 (25.9)	262 (26.0)	301 (25.0)	301 (25.1)	254 (25.0)
WA	531 (10.4)	98 (10.0)	118 (11.0)	118 (10.1)	143 (10.0)
Location					
Capital city	3148 (61.7)	654 (65.6)	772 (65.0)	773 (61.5)	637 (54.9)
Regional/remote	1952 (38.3)	370 (34.4)	428 (35.0)	428 (38.5)	508 (45.1)

^a^ Unweighted.

^b^ Weighted for age, gender and state.

### 3.2 Self‐Rated Health Status

In 2021, three‐quarters of Australians rated their health as either good (*n* = 1678, 32.9%) or very good to excellent (*n* = 2353/5100, 46.1%). However, across the five surveys, self‐rated health status significantly declined, *F*(1,9667) = 67.42, *p* < 0.001); in 2021 and 2018, an average of 45.2% (*n* = 2767/6124) of Australians rated their health as very good to excellent, compared with 56.3% (*n* = 1994/3544) of Australians across the 2012, 2010 and 2008 Menzies‐Nous surveys. Across the five surveys, younger Australians (aged 18–44 years) (*n* = 2792/4731, 59.0%) self‐rated their health higher than older Australians (aged ≥ 45 years) (*n* = 1966/4930, 39.9%) (*χ* ^2^(1, *N* = 9661) = 353.73, *p* < 0.001) and Australians residing in cities (*n* = 3096/6009, 51.5%) rated their health higher than Australians in regional or remote regions (*n* = 1665/3657, 45.5%) (*χ* ^2^(1, *N* = 9666) = 32.68, *p* < 0.001). There were no significant gender differences in self‐rated health status.

### 3.3 Visits to General Practice

In 2021, most participants reported that they always try to see the same GP (*n* = 3669/5100, 71.9%) or go to the same GP practice (*n* = 909/5100, 17.8%). For 2021 and 2018, reported efforts to see the same GP (*n* = 4430/6124, 72.3%) were higher than in previous years (*n* = 2173/3547, 61.3%), as determined by chi‐squared analysis (*χ* ^2^(3, *N* = 9671) = 228.99, *p* < 0.001). Across the five surveys, older Australians (aged ≥ 45 years) (*n* = 3834/4931, 77.8%) were more likely to report trying to see the same GP than younger Australians (aged 18–44 years) (*n* = 2767/4733, 58.5%) (*χ* ^2^(3, *N* = 9664) = 415.23, *p* < 0.001). There were no significant gender or geographic location differences identified for this item.

### 3.4 Overall Views Towards the Healthcare System

**Figure 1:**
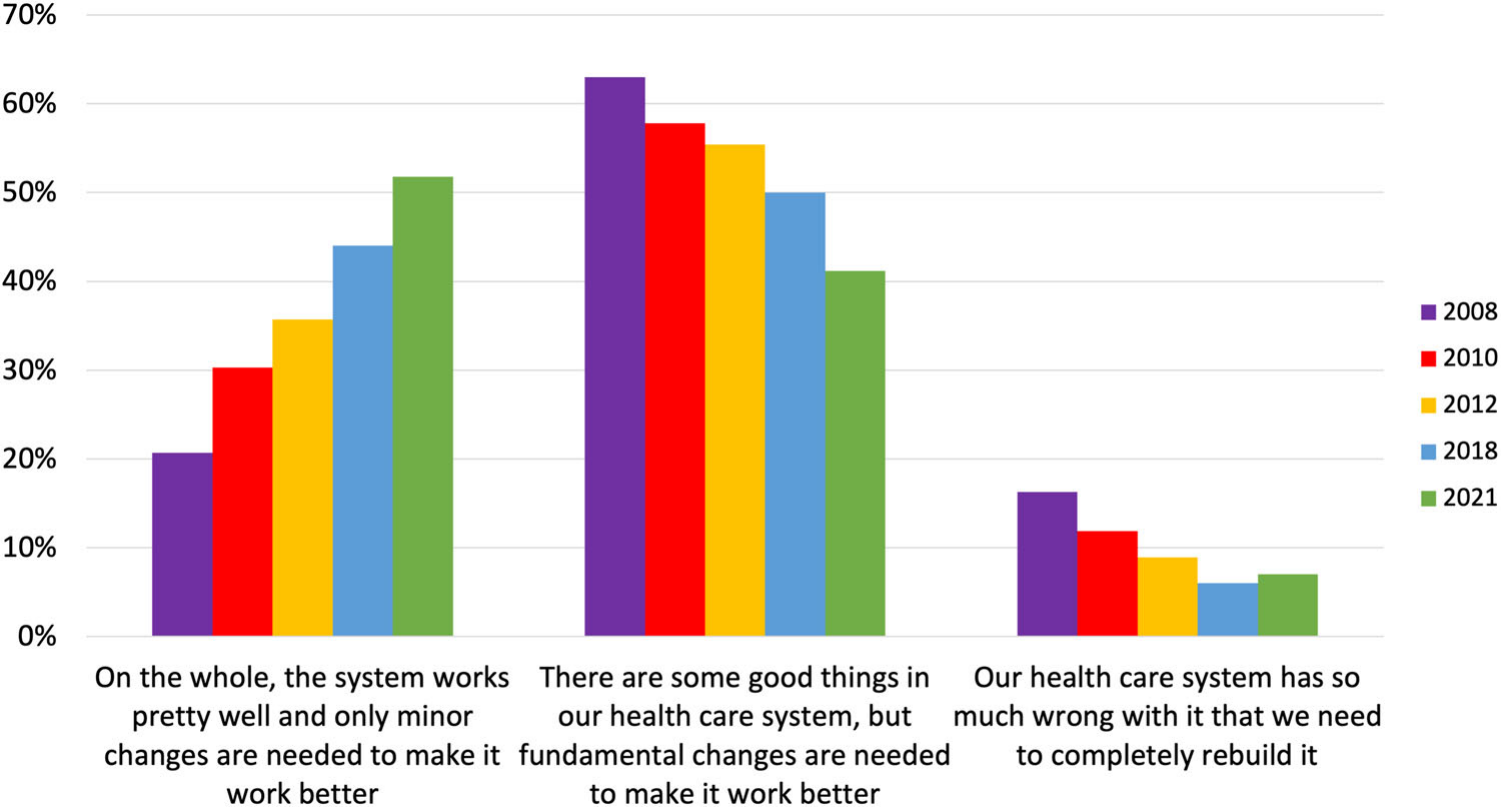
Australians' views of the healthcare system 2008 to 2021.

In 2021, under half of participants reported that ‘there are some good things in the Australian healthcare system, but fundamental changes are needed to make it work better’ (*n* = 2089/5100, 41.0%). However, there has been a significant change since 2008, with a greater proportion of respondents viewing the Australian healthcare system more positively (*χ* ^2^(8, *N* = 9645) = 487.63, *p* < 0.001) (also see Figure 1). Across the 2021 and 2018 Australian Consumer Sentiment surveys, over half of participants (*n* = 3118/6123, 50.9%) identified that the health ‘system works pretty well, and only minor changes are needed to make it work better’, up from only 30% across the earlier Menzies‐Nous surveys (*n* = 1065/3521) (*χ* ^2^(2, *N* = 9644) = 397.45, *p* < 0.001). Over the five surveys, views on the healthcare system differed by age with participants aged 25–64 years being more likely to identify a need for ‘fundamental changes’ to be made (*n* = 3368/6652, 50.6%), in comparison to those in the youngest and oldest age groups (18–24 years, and 65+ years) (*n* = 1266/2984, 42.4%) (*χ* ^2^(2, *N* = 9636) = 71.91, *p* < 0.001). A higher proportion of women identified a need for fundamental changes to be made (*n* = 2567/4897, 52.4%) compared to men (*n* = 2057/4700, 43.8%) (*χ* ^2^(2, *N* = 9597) = 102.93, *p* < 0.001). Finally, a greater proportion of respondents living in regional or remote regions identified the need to ‘completely rebuild’ healthcare (*n* = 390/3651, 10.7%) compared to Australians living in major cities (*n* = 431/5992, 7.2%) (*χ* ^2^(2, *N* = 9643) = 40.73, *p* < 0.001).

### 3.5 Confidence in the Australian Healthcare System

**Figure 2:**
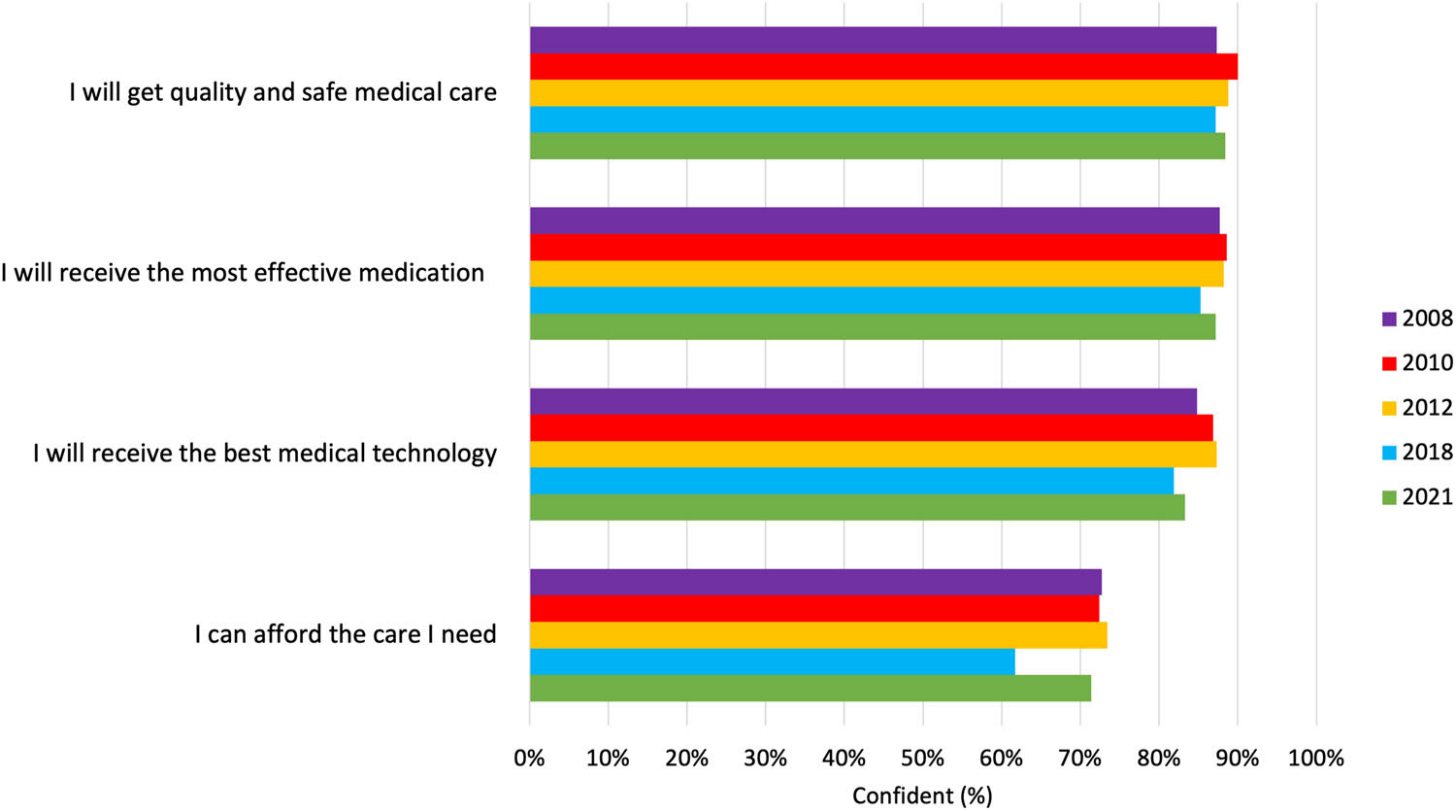
Australians' level of confidence in the healthcare system.

In 2021, over four‐fifths of participants identified that they were ‘somewhat’ or ‘very confident’ that upon becoming seriously ill they would receive quality and safe medical care (*n* = 4510/5100, 88.4%); the most effective medication (*n* = 4449/5100, 87.2%); and the best medical technology (*n* = 4249/5100, 83.3%). Around 70% of respondents expressed confidence that they would be able to afford the care needed (*n* = 3642/5100, 71.4%). Results for 2021 were similar and not significantly different to the confidence reported for the former four surveys, as determined by chi‐squared analysis (also see Figure 2). Across the five surveys, men were significantly more likely than women to report confidence in getting quality and safe medical care (men: *n* = 4289/4700, 91.3%; women: *n* = 4236/4897, 86.5%) (*χ* ^2^(1, *N* = 9597) = 54.61, *p* < 0.001), receiving the most effective medication (men: *n* = 4216/4684, 90.0%; women: *n* = 4163/4868, 85.5%) (*χ* ^2^(1, *N* = 9552) = 44.69, *p* < 0.001), receiving the best medical technology (men: *n* = 4081/4690, 87.0%; women: *n* = 4013/4879, 82.3%) (*χ* ^2^(1, *N* = 9569) = 41.63, *p* = < 0.001) and being able to afford care (men: *n* = 3540/4689, 75.5%; women: *n* = 3256/4882, 66.7%) (*χ* ^2^(1, *N* = 9571) = 90.01, *p* < 0.001). Older participants were significantly more confident than younger participants in getting quality and safe medical care (65+ years: *n* = 1682/1825, 92.2%; 18–64 years: *n* = 6870/7813, 87.9%) (*χ* ^2^ (1, *N* = 9638) = 26.53, *p* = < 0.001), receiving the most effective medication (65+ years: *n* = 1672/1814, 92.2%; 18–44 years: *n* = 6739/7781, 86.6%) (*χ* ^2^ (1, *N* = 9595) = 42.10, *p* = < 0.001) and receiving the best medical technology (≥ 65 years: *n* = 1634/1818, 89.9%; 18–44 years: *n* = 6493/7793, 83.3%) (*χ* ^2^(1, *N* = 9613) = 48.60, *p* < 0.001). Participants living in cities were more likely to report confidence in being able to afford care (*n* = 4352/5984, 72.7%) compared to those living in regional or remote regions (*n* = 2476/3634, 68.1%) (*χ* ^2^(1, *N* = 9618) = 23.16, *p* < 0.001). No other significant geographic location differences were found.

### 3.6 Additional Questions Specific to the 2021 Survey

In 2021, since the COVID‐19 pandemic, over two‐thirds of respondents (*n* = 3949/5100, 77.4%) reported that their confidence in the Australian healthcare system had either stayed the same (*n* = 2433/5100, 47.7%) or even increased (*n* = 1516/5100, 29.7%). Men were more likely to report increased confidence in the Australian healthcare system since COVID‐19 (*n* = 860/2476, 34.7%) than women (*n* = 645/2577, 25.0%) (*χ* ^2^(1, *N* = 5053) = 56.86, *p* < 0.001). Younger Australians (18–44 years) were more likely to report increased confidence since COVID‐19 (*n* = 959/2467, 38.9%) compared with older Australians (≥ 45 years) (*n* = 556/2632, 21.1%) (*χ* ^2^(1, *N* = 5099) = 192.08, *p* < 0.001). However, respondents from cities were less confident, with a higher proportion reporting decreased confidence in the Australian healthcare system since COVID‐19 (*n* = 764/3148, 24.3%) than participants living in regional or remote regions (*n* = 387/1952, 19.8%) (*χ* ^2^(2, *N* = 5100) = 14.61, *p* < 0.001).

**Figure 3:**
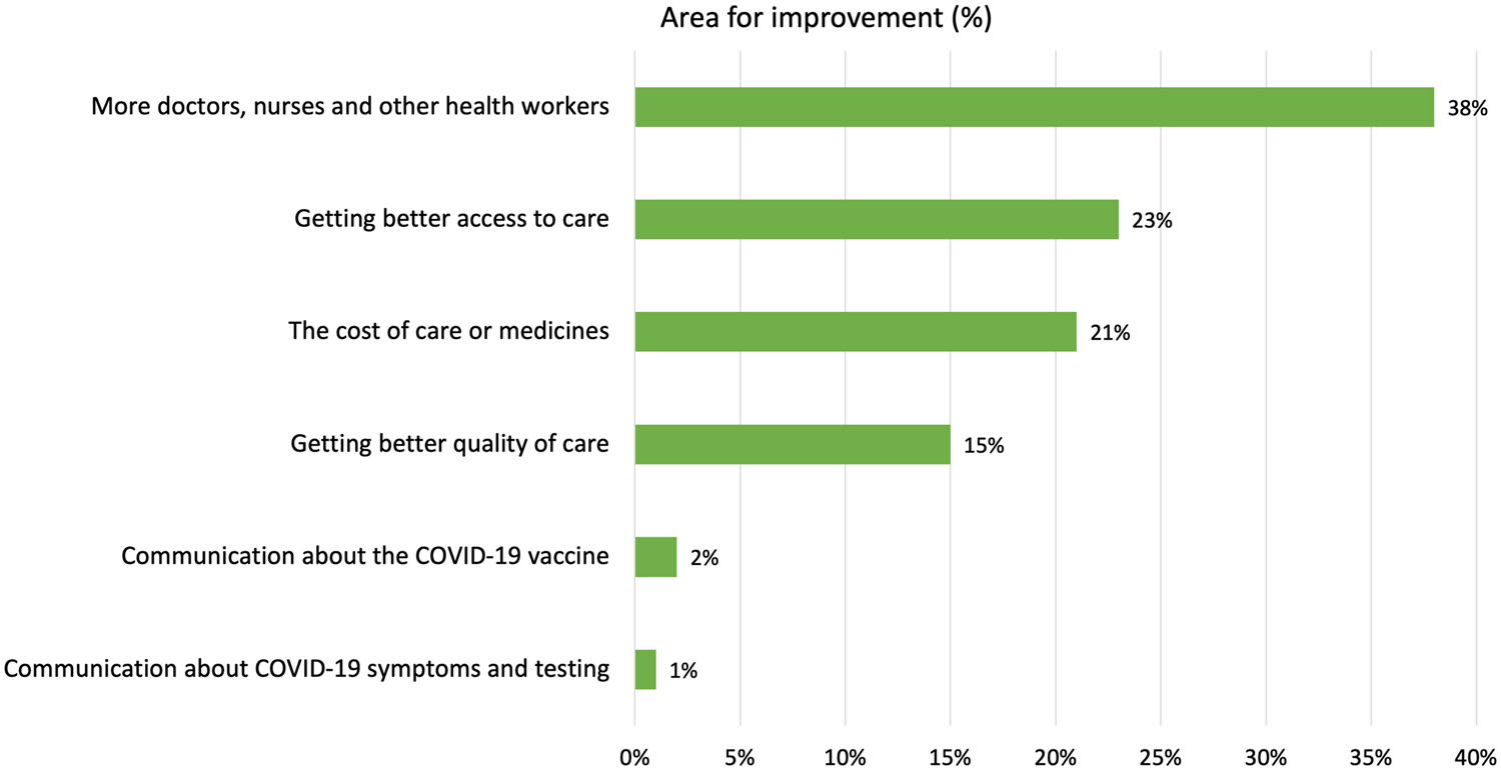
Area of the health system most urgently in need of improvement from the 2021 Australian Health Consumer Sentiment Survey.

In 2021, participants identified that the greatest improvement needed to the healthcare system is the need for ‘more doctors, nurses and other healthcare workers’ (*n* = 1350/3576, 37.8%); ‘better access to care’ (*n* = 809/3576, 22.6%); and the ‘cost of care or medicines’ (*n* = 757/3576, 21.2%). Figure 3 provides a summary. Participants living in regional or remote regions were significantly more likely to report the need for more doctors, nurses and other healthcare workers (*n* = 590/1385, 42.6%) compared to Australians living in cities (*n* = 760/2192, 34.7%) (*χ* ^2^(5, *N* = 3577) = 33.26, *p* = < 0.001). Older Australians (aged 65+ years) were significantly more likely to report the need for healthcare workers (65+ years: *n* = 503/770, 65.3%; 18–64 years: *n* = 847/2807, 30.2%) (*χ* ^2^(5, *N* = 3577) = 338.59, *p* < 0.001), whereas younger Australians (18–44 years) were more likely to report the need for better access to care (18–44 years: *n* = 534/1694, 31.5%; ≥ 45 years: *n* = 275/1883, 14.6%) and better quality of care (18–44 years: *n* = 372/1694, 22.0%; ≥ 45 years: *n* = 175/1883, 9.3%) (*χ* ^2^(5, *N* = 3577) = 462.54, *p* < 0.001).

## 4 Discussion

This study offers insight into the views held by Australians about the health system during the COVID‐19 pandemic, and cross‐sectional differences across five surveys spanning 13 years. In 2018 and 2021, mostly positive views towards the Australian health system were expressed by the Australian public. These views have improved over time. Over half of Australians viewed their healthcare system positively in 2018 and 2021; a significant improvement from only 30% across earlier surveys in 2008, 2010 and 2012.

Looking at international comparison surveys, the 2020 Ipsos Global Health Monitor ranked Australia first for healthcare satisfaction out of 27 countries, with Australians reporting the highest satisfaction when it comes to its healthcare system [9]. However, in the same survey, Australia ranked seventh for the provision of affordable healthcare [9]. Our study also pointed to concerns around the cost of healthcare in Australia. Across all five surveys since 2008, the vast majority of respondents reported confidence that they would receive quality and safe care upon becoming ill; however, only around two‐thirds expressed confidence that they would be able to afford needed care.

Notably, Australians living in cities were more likely to report confidence in being able to afford care, compared to those living in regional or remote areas. This may be because of limited publicly funded health services in regional centres and remote towns with specialists and GPs in these areas being more likely to charge co‐payments from patients in addition to the fees covered under Medicare [34]. Indeed, worry among the Australian public in relation to healthcare affordability is consistent with past research [35, 36] on out‐of‐pocket healthcare expenses, and warrants further research.

The results of this study demonstrate a deterioration in self‐rated health status over the 13‐year period from 2008. Less than half of Australians self‐rated their health as very good to excellent in 2018 and 2021; compared with 56% across the 2008, 2010 and 2012 surveys. However, previous research on longitudinal self‐reported health trajectories suggests that this finding may reflect a decline in subjective perceptions rather than an actual deterioration in health status over time [37]. Nevertheless, there is substantial international evidence that people residing in regional or remote areas are more likely to have poorer health status, including higher rates of chronic conditions [38]. A large study from the United States identified that rural/urban differences in health could be explained almost entirely by rural structural disadvantage, that is, through reduced investment in health infrastructure, access to healthcare workers and transportation systems [39]. The swift expansion of telehealth services amid the COVID‐19 pandemic has the potential to overcome much of this structural disadvantage; however, additional research is required to more fully understand the enduring consequences of telehealth adoption in regional and remote areas.

Across 2021 and 2018, around three‐quarters of Australians reported that they always try to see the same GP; this has increased from the former Menzies‐Nous surveys [23, 24, 25] and suggests that the Australian public seek a consistent relationship and continuity of care from their GP. In Australia, patients are not bound to be registered with a general practice and may choose to see multiple GPs in multiple locations according to personal preferences [40]. However, recent policy suggestions have endorsed a shift towards a model of voluntary enrolment with GPs (now known as MyMedicare) on the grounds that ‘having a regular GP is beneficial for patient outcomes, patient experience and value for the system’ [41, p. 48]. Our findings broadly reflect growing recognition of the value of continuity of care amongst the Australian public, particularly for older people who are more likely to have chronic health conditions [42]. However, the vast majority of GPs in Australia are not in favour of voluntary patient enrolment with concerns that MyMedicare will reduce funding and give the government more control over primary care delivery [43].

There were several other key areas of improvement identified by the Australian public, most notably the need for more doctors, nurses and other health workers. This concern is reflected in international research which has predicted a worldwide shortage of 10 million healthcare workers by 2030 [44]. In Australia, an estimated 300,000 additional workers will be needed in the healthcare sector over the next 5 years [45]. Australians living in regional or remote regions were more likely to report the need for more healthcare workers. Australians living in regional and remote regions were also more likely to indicate that the healthcare system needed to be completely rebuilt compared to Australians living in major cities. Challenges with attracting and retaining healthcare workers are being felt across Australia [46] and internationally [47]. Suggested solutions to GP shortages often include initiatives to improve integrated team‐based care and upskilling nurses and allied health professionals [48]. Other key areas for improvement identified in our study included securing better access to care and lowering the cost of care and medicines. These identified areas of improvement highlight key changes needed in the health system to meet public needs and experiences.

In this study, younger Australians (18–44 years) had significantly less confidence in receiving high‐quality and safe care, effective medicines and the best medical technologies. These results align with a recent report from the Australian Bureau of Statistics which shows that young people aged under 25 years had longer waiting times to see a GP or a specialist doctor and people aged 25–34 years delayed or did not see a medical specialist when needed because of cost [49]. Access barriers to healthcare for young people have been recognised and include cost of care and limited access to age‐appropriate services and practitioners skilled in caring for young people [50].

Women were also less likely than men to express confidence in receiving and being able to afford care; while also reporting poorer health status than men. These findings align with recent Australian data showing that women have longer waiting times to see GPs and specialist doctors than men [49]. Gender bias in healthcare access and healthcare quality has long been reported [51]. For example, The Lancet Women and Cardiovascular Disease Commission recently highlighted the ongoing gaps in ‘research, prevention, treatment and access to care for women’ and called for action to address gender disparities in healthcare [52]. In addition, the recent rise in cost of living including increases in healthcare costs [53, 54, 55] are disproportionately impacting women's [56, 57] and young people's health [54, 56, 57]. Decisive responses are needed from government and health insurers to support affordable access to healthcare now to avoid additional increased burdens on health systems in the future.

Over two‐thirds of Australians reported that their confidence in the Australian healthcare system had either stayed the same or increased since the advent of COVID‐19. This likely reflects Australia's success in containing the COVID‐19 pandemic, with Australia experiencing lower COVID‐19 cases and deaths than many countries [3]. ‘Flattening the curve’ became a key public health message in Australia and had a demonstrable effect on public behaviour, along with the use of collective messaging (e.g., ‘we are in this together’) to build shared identity as a driver of social influence [58]. However, older Australians, women and respondents residing in cities were less likely to report increased confidence since COVID‐19 reflecting research showing that Australia's lockdown measures adversely affected health and access to healthcare services, particularly for women [59, 60] and older Australians [61], and for those living in two of Australia's most populous cities, Melbourne and Sydney, who underwent the harshest and longest lockdowns.

### 4.1 Strengths and Limitations

This study compared the perceptions of representative samples of Australians across five time points from 2008 to 2021, with such data being rarely available elsewhere [12]. Health consumer representatives from the Consumers Health Forum of Australia co‐designed the survey and were involved in the analysis and interpretation of the results [12, 22]. However, there were some inconsistencies in methods between surveys. For the former Menzies‐Nous surveys, survey responses were collected data via Computer‐Assisted Telephone Interviewing (CATI) methods, rather than online survey methods [12, 23, 24, 25]. Although this change of methods reflects a major global shift in survey and market research towards online panel surveys, online consumer panels can be subject to bias and may not be truly representative of the general population [62]. For example, online surveys may miss individuals who do not have access to or regularly use technology (e.g., older individuals). Finally, we were unable to establish a response rate because of the sampling process applied to the established panel and we have no details on non‐respondents.

### 4.2 Implications and Conclusions

The findings of this study provide important views and experiences of the Australian public about their health system, at a time when the COVID‐19 pandemic was still very present, and with differences in views examined spanning 13 years. As in previous analyses of this survey [12, 22], this survey provides important findings regarding changing public perceptions of the health system that are valuable to policymakers, health services and health providers.

Overall, comparisons across surveys have shown that Australians' perceptions of their healthcare system have continued to improve, despite persistent disruptions to the Australian healthcare system caused by the COVID‐19 pandemic. Problem areas have been consistently identified, including the need for more health workers. Additionally, barriers to access and cost of care or medicines continue to be a concern and the recent increases in the cost of living may further impact healthcare access, experiences and opinions. Future surveys should serve to monitor any potential responses to these identified concerns.

## Author Contributions

**Louise A. Ellis:** conceptualisation, writing–original draft, methodology, writing–review and editing, formal analysis, visualisation, data curation. **Genevieve Dammery:** project administration, writing–review and editing, visualisation. **James Gillespie:** conceptualisation, writing–review and editing, methodology. **James Ansell:** conceptualisation, funding acquisition, writing–review and editing, methodology, data curation. **Leanne Wells:** conceptualisation, funding acquisition, methodology, writing–review and editing, resources. **Carolynn L. Smith:** project administration, writing–review and editing, methodology. **Shalini Wijekulasuriya:** writing–review and editing, project administration, visualisation. **Jeffrey Braithwaite:** supervision, writing–review and editing. **Yvonne Zurynski:** conceptualisation, methodology, writing–review and editing, supervision.

## Ethics Statement

This study was approved by the Macquarie University Human Research Ethics Committee (Ref. No.: 52021367031878). All participants provided online informed consent to participate.

## Conflicts of Interest

The authors declare no conflicts of interest.

## Supporting information

Supporting information.

## Data Availability

The data that support the findings of this study are available upon request from the corresponding author. The data are not publicly available due to privacy or ethical restrictions.
