# Supplementary material for: Public Perceptions of the Australian Health System During COVID‐19: Findings From a 2021 Survey Compared to Four Previous Surveys
Source: Health Expect. 2024 Jul 11;27(4):e14140. doi: 10.1111/hex.14140 (PMC11239533; doi:10.1111/hex.14140)
Supplement: Supplementary file 1 — Supporting information. [file HEX-27-e14140-s001.docx]

## Appendix 1

Summary results from 4 sets of dummy variables

| Item | Age 1 | | Age 2 | | Age 3 | | Age 4 | |
| --- | --- | --- | --- | --- | --- | --- | --- | --- |
|  | % | *X^2^* | % | *X^2^* | % | *X^2^* | % | *X^2^* |
| Self-rated health status (very good to excellent) | 60.9 v 47.7 | 72.00* | 39.9 v 59.0 | 353.73** | 34.8 v 52.6 | 189.04* | 44.9 v 51.2 | 32.2* |
| Always try and see the same GP | 58.7 v 69.6 | 64.50* | 77.8 v 58.5 | 415.23** | 86.9 v 64.0 | 363.39* | 75.9 v 64.9 | 118.64* |
| Fundamental changes are needed to make the health system better | 44.6 v 48.6 | 22.27* | 47.4 v 48.9 | 26.72* | 41.1 v 49.7 | 46.51* | 42.4 v 50.6 | 71.91** |
| Quality and safe medical care (somewhat confident to very confident) | 87.9 v 88.9 | 1.02 | 89.5 v 87.9 | 6.44 | 92.2 v 87.9 | 26.53** | 90.5 v 87.9 | 13.46* |
| Receive the most effective medication (somewhat confident to very confident) | 84.4 v 88.1 | 12.68* | 89.0 v 86.3 | 16.10* | 92.2 v 86.6 | 42.10** | 89.2 v 87.0 | 8.94* |
| Receive the best medical technology (somewhat confident to very confident) | 80.9 v 85.0 | 13.22* | 86.4 v 82.6 | 27.21* | 89.9 v 83.3 | 48.60** | 86.4 v 83.7 | 11.22* |
| Able to afford care (somewhat confident to very confident) | 69.8 v 71.1 | 0.82 | 69.9 v 72.1 | 5.23 | 72.8 v 70.6 | 3.66 | 71.7 v 70.7 | 0.99 |
| Increased confidence since COVID-19 | 37.0 v 28.7 | 17.54* | 21.1 v 38.9 | 192.08** | 18.9 v 32.5 | 73.7* | 25.6 v 31.7 | 20.11* |
| More doctors, nurses and other health workers | 20.6 v 39.9 | 61.05* | 53.2 v 20.6 | 462.54** | 65.3 v 30.2 | 338.59* | 50.1 v 31.8 | 122.37* |

Note. Age 1: 1 = <24 years, 0=other; Age 2: 1 = 45+ years, 0 = other; Age 3: 1 = 65+ years, 0 = other; Age 4: 1 = <24 years or >65 years, 0 = other

*p < .001; **most significant dummy variable in set
